# Early Postnatal Protein-Calorie Malnutrition and Cognition: A Review of Human and Animal Studies

**DOI:** 10.3390/ijerph8020590

**Published:** 2011-02-23

**Authors:** Maria Fernanda Laus, Lucas Duarte Manhas Ferreira Vales, Telma Maria Braga Costa, Sebastião Sousa Almeida

**Affiliations:** 1 Laboratory of Nutrition and Behavior, Department of Psychology and Education, Faculty of Philosophy, Sciences and Letters of Ribeirão Preto, University of São Paulo, Av. Bandeirantes, 3900, 14040-901, Ribeirão Preto, SP, Brazil; E-Mails: fernandalaus@pg.ffclrp.usp.br (M.F.L.); lucasvales@yahoo.com.br (L.D.M.F.V.); 2 Nutrition Course, University of Ribeirão Preto, Av. Costábile Romano, 2.201, Bloco U, 14096-900, Ribeirão Preto, SP, Brazil; E-Mail: tbraga@unaerp.br

**Keywords:** malnutrition, development, protein deprivation, cognition, environment

## Abstract

Malnutrition continues to be recognized as the most common and serious form of children’s dietary disease in the developing countries and is one of the principal factors affecting brain development. The purpose of this paper is to review human and animal studies relating malnutrition to cognitive development, focusing in correlational and interventional data, and to provide a discussion of possible mechanisms by which malnutrition affects cognition.

## 1. Introduction

Malnutrition can be defined as “an imbalance between the supply of protein and energy and the body’s demand for them to ensure optimal growth and function” [1]. Although it includes a state of deficiency or excess of energy, protein, and other nutrients, the present review will discuss only studies designed to investigate protein and/or calorie deficiency, which includes three main clinical syndromes: kwashiorkor, marasmus and marasmic kwashiorkor.

Nutrition early in life represents one of the major important variables directly related to formation, growth, and functional organization of the organism. An inadequate diet during the first period of life has the potential to influence adversely the development of the brain, which is genetically programmed to grow more quickly than the rest of the body, resulting in changes to its structure and hence functioning. Malnutrition has been recognized to cause reductions in the numbers of neurons, synapses, dendritic arborization, and myelination, all of which result in decreased brain size. Also, the cerebral cortex is thinned and brain growth slowed. All these central nervous system alterations are associated with delays in motor and cognitive functions, such as attention deficit disorder, impaired school performance, decreased IQ scores, memory and learning deficiencies, and reduced social skills.

Historically, the effects of malnutrition on cognitive development have been studied in both humans and animals, although the animal studies began earlier than human studies and are more abundant in the literature. Research involving humans is mainly conducted in children in developing countries and comprises correlational and interventional studies. On the other hand, experimental animal studies are essential to understand the mechanisms involved in these relationships, since some points cannot be elucidated in humans by several reasons (*i.e.*, ethics, economical).

It is important to highlight that there are few areas on the field of malnutrition studies where it is possible to establish a link between basic research conducted with animals and that more applied studies conducted with human subjects. However, it is possible to compare studies with animals and humans malnourished in early life if we focus in the basic psychological processes involved in the cognitive development (*i.e.*, learning, memory and emotion), so the purpose of this paper is to present studies conducted with humans and animals relating malnutrition to cognitive development in an attempt to provide evidences for a discussion of the possible biological and/or environmental mechanisms by which malnutrition affects cognition.

### Review Methodology

The studies published in the literature and utilized for analysis in the present review were identified using a combination of techniques aimed at finding the relevant articles on the field. Search techniques included keyword and author searches using the National Library of Medicine’s PUBMED database, and a survey of citations included in recent research and review articles. Abstracts were not included. Keywords used were: malnutrition, development, protein deprivation, cognition, environment, rodents, children, nutritional supplementation, psychosocial stimulation, learning and memory.

The approach taken in this review took into account correlational studies, nutritional supplementation studies and/or psychosocial stimulation studies, and experimental studies. Since this is a comprehensive field, we decided to restrict our search to studies relating malnutrition and cognitive development in humans and rodents only, excluding those that did not give emphasis to cognitive processes.

After an initial search for studies on malnutrition and cognition in animal models, we decided to restrict studies to rodents. The experimental animal models of research about the effects of malnutrition on cognitive development are more numerous in rodents. Most studies of malnutrition in animal models use rodents due to the ease of breeding, maintenance and treatment, in addition to the short life cycle of those subjects, as well as, for ethical and economic reasons. In summary, studies with rodent have well-established and validated models for the study of behavior and cognition in malnourished organisms, enabling a saving of time and financial resources quite significantly.

All papers resulting from that selection were used in the present review and the papers are summarized Table 1 (18 human studies that matched the review criteria) and Table 2 (37 animal studies that matched the review criteria).

### 2. Human Studies

Besides being considered a major cause of death among children, nutritional inadequacy is one of the principal factors affecting the development of the brain. Several studies conducted in Barbados [2,3], India [4,5], Philippines [6], Peru [7] and Brazil [8] have found that severe postnatal protein-calorie malnutrition negatively influences cognitive development. These correlational studies have demonstrated significant associations between malnutrition and deficits in analytic and reasoning skills [6]; language (*i.e.*, verbal fluency, vocabulary, verbal comprehension) [4,5,8]; visuospatial working memory [5,8]; visuospatial functions, attention and learning [5]; intellectual performance (including IQ) [2,7] and educational achievement [3]. Obviously, the severity and duration of the nutritional insult determines the effects on cognitive development, but it is known that these complications can be observed even in children who suffer from mild insults over extended periods.

Based on these previously and currently evidences on the relationship between malnutrition and cognition, several studies have being conducted in developing countries since the late 1970s to evaluate the possible effects of dietary interventions programs (supplementation studies) on children’s cognitive performance. One classical example in the literature is the longitudinal study of Chavez *et al.* [9], carried out with two groups of 17 mother-child pairs, in a poor rural Mexican community, to evaluate the effects of nutritional supplementation on infants’ physical, mental, and social development. In this study, one group was not supplemented and followed the usual feeding habits of the community, while in the other group mothers were supplemented daily with 205 calories and 15 grams of protein during pregnancy and 305 calories and 15 grams of protein during lactation. Between the 12th and 16th week of life the supplemented infants began to receive*, ad libitum*, whole cow’s milk and prepared baby food in quantities sufficient to maintain adequate rates of growth. After a few weeks, supplemented children developed a different pattern of interaction with both the mother and the environment, e.g., they were sleeping less, refusing to use the cradle and to be carried wrapped up, and playing more. By 9 months, the supplemented children were receiving more stimulation and rewards, from both parents. At 18 months of intervention, the mothers of supplemented children displayed more complex interactions with their children, who were more restless, playful, demanding and disobedient than nonsupplemented ones.

These results suggest that supplementation was effective in elevating the children’s activity level and influencing the behavior patterns within the family, with the more active children eliciting greater stimulation from their parents. Another study, conducted in Guatemala by Pollitt and colleagues [10], demonstrated that protein supplementation during early childhood improved psychoeducational performance. Children were exposed to either a protein or an energy supplement and those who received the protein one scored significantly higher on tests of knowledge, numeracy, reading and vocabulary than subjects exposed only to energy supplementation. Interesting, of all children who received supplements, 130 females were evaluated 20 years later [11] and the authors found that those who were exposed to the protein supplement had better educational achievement than the ones in the energy group, showing that the benefits of supplementation continue through later life. Whaley and her co-workers [12] carried out a study in Kenya with 555 malnourished school children and demonstrated that supplementation with animal source of food improved the ability to organize perceptual detail; reasoning by analogy and form comparisons; and arithmetic ability but not verbal comprehension, indicating that the intervention successfully improved cognitive performance, however it was not equivalent across all domains of cognitive functioning.

Studies like these provide evidence that food supplements can improve children’s development and show concurrent benefits to cognitive ability. However, still remains unclear if malnutrition *per se* contributes more or less to impair cognitive development of previously malnourished children than do adverse social and environmental conditions. That way, the presumed link between malnutrition and cognitive development are challenged by the findings of numerous investigations of the influence of the physical and social environment on the child’s cognitive functioning.

A study conducted in four Guatemalan villages [13,14] evaluated the relations between nutrition and cognition, taking into account the social environment. The authors previously had found that both nutritional and social factors are related to cognitive development, but the magnitude of the associations between them varies markedly by cognitive domain [15]. Results demonstrated significant correlations between cognitive performance (language facility, short-term memory for numbers and perceptual analysis) and nutrition, as well as social-environment factors (quality of house, mother’s dress and task instruction), but nutrition explained better the worse cognitive performance than the social-environmental factor alone.

Two other experimental studies were carried out in Colombia to evaluate the effects of a program of treatment combining nutritional supplementation and/or educational features on children’s cognitive development. McKay *et al.* [16] tested an intervention program that included nutritional supplementation (that provides a minimum of 75% of recommended daily protein and calorie allowances by means of low-cost foods available commercially, supplemented with vitamins and minerals, and offered *ad libitum* three times a day); health care and an educational treatment (designed to develop cognitive processes and language, social abilities, and psychomotor skills) on language usage, immediate memory, manual dexterity and motor control, information and vocabulary, quantitative concepts, spatial relations, and logical thinking, with a balance between verbal and nonverbal production. The authors concluded that environmental deprivation retards general cognitive development and that combined nutritional, health, and educational treatments can prevent large losses of potential cognitive ability, with significantly greater effect the earlier the treatments begin. Similarly, Waber and colleagues [17] tested the effects of nutritional supplementation and/or a maternal education program on children’s cognitive competence. Two groups received only the nutritional supplement in different periods, one group received only maternal education and another one received both. The results showed that children who received nutritional supplementation and, to a lesser extent, those who received maternal education, performed better than children who did not receive those treatments; but the effects of the two treatments appeared to be independent, each affecting different aspects of performance. Nutritional supplementation affected primarily motoric functions while maternal education had its clearest impact on language.

The effects of nutritional supplementation and early childhood psychosocial stimulation were also evaluated in Jamaican children [18–21]. The authors followed up 127 malnourished children who received nutritional supplementation with or without psychosocial stimulation at 9–24 months of age during 2 years. Supplementation comprised 1 kg of milk-based formula per week, and stimulation comprised weekly 1-h home visits by community health workers, with the objective of improving mother-child interactions through play. Emphasis was placed on improving the teaching techniques of the mothers and the quality of verbal interactions between mother and child, and concepts of color, shape, size, number, and position were taught. The first evaluation started right after the intervention ceased and the results showed that the development of children who received both treatments caught up with that of the non-stunted children [18]. At ages 7 [19] and 11 years [20], children were visited again. At both ages, malnourished children originally assigned no intervention had poorer intelligence levels and had poorer cognitive function than the children who were not stunted. Furthermore, at age 11 years, the malnourished children were not doing as well at school, but the ones who received stimulation displayed significant benefits to cognition (IQ). At 17 years of age, the authors recorded no significant effects of nutritional supplementation; however the stimulation resulted in higher full scale IQ scores and better performances in verbal, vocabulary, and reading tests [21].

Although the human studies are clear on the effects of early protein-calorie malnutrition on behavior and cognition, with a special role for environmental factors concurrent to nutrition deprivation, it also clear that we continue depending on experimental animals studies to understand the nutritional-environmental-behavioral relationships. As previously commented, certainly, some questions on the area can not be answered without animal studies that, by ethical, economic and time reasons, are not possible to be conducted using human subjects.

## 3. Animal Studies

According to behavioral data reported in the last decades, most evident changes observed in prenatal and postnatal malnourished rats leads to increased motivation for food or water in response to privation [22,23], emotional reactivity and sensitivity to aversive or painful stimuli [24–28], reduction in cognitive flexibility [59,60] and learning and memory impairments [29–35]. Behavioral changes reported are due to functional or morphological alterations, particularly in the neocortex and hippocampal formation [61].

The time length and magnitude of nutritional insult is directly related to the severity and duration of its effects on body development and on behavioral changes. Protein and/or protein-calorie malnutrition are the most commonly used experimental models of early malnutrition in animal studies. Most of these studies are developed in rodents. Like other mammalian species studied, rats exhibit a sequence of development of the central nervous system similar to humans with specific differences in relation to the maturing brain in each species [62]. The short life cycle of rodents (especially rats) make it possible to conduct studies in several generations of the animals in a brief time in the laboratory, as well as, to develop animal experimental models to study the effects of inter-generation malnutrition on biological and behavioral parameters.

There are several techniques of experimental malnutrition developed to be used in rodents. The techniques that induce malnutrition during the post-natal life (mainly during the lactation period) produce significant impairments in the development of the animals with a great decrease in the growth of pups that will remain even after a prolonged period of nutritional recovery. The mainly cause for this important growth retardation is the reduction in the quantity and quality of the mother’s milk suckled by pups [36–38].

Protein and/or protein-calorie malnutrition during the lactation period can be produced by a low-protein diet (reduction of the protein amount present in diet composition); reduction or inadequate amount of diet available; large litters (malnutrition by increasing litter’s size) and malnutrition by separation (removal of the pups from the dams for a period o time during the day). All of these techniques are efficient to produce significant effects both on physical and behavioral developments in rats, as demonstrated by Hernandes and her co-workers [39].

Protein or protein-calorie malnutrition imposed during lactation changes the quantity and quality of mother-pup interactions and may lead to long-lasting adverse effects on the behavior of rats in the adult life [40–42]. Reported data have been shown that malnourished mothers spend more time in the nest [41], take longer to gather the pups in the pup retrieval test [40] and change the eating pattern [43]. Like the mothers, the malnourished pups also presented changes in the behavior as compared to well-nourished pups, mainly by expressing more time in the nest, less time exploring the environment, less time separated in groups and more time in ultrasonic calls [42,44].

Although many animal techniques of early malnutrition have been studied and widely described in the past 50 years, there are few studies of postnatal protein malnutrition and its effects on cognition, learning and memory processes. For this reason, we will briefly present and discuss some studies from the last 10 years which presents new and relevant discoveries about the consequences of postnatal protein malnutrition on cognition, learning and memory.

A study conducted in our group showed that rats malnourished in early life presented significant alterations in social interaction behaviors in childhood and these behaviors were related to dominance behaviors in adulthood [45]. During lactation phase the litters were fed diet containing 16% protein (well-nourished) or 6% protein (malnourished). The animals were divided into three groups: well-nourished (W), malnourished (M) and previously malnourished (PM). From weaning to the end of behavioral tests W group and PM were fed a commercial lab chow diet and M group was maintained on a 6% protein diet. Pairs of male rats of same diet conditions were tested, at different ages, for three consecutive days. According to analyzed data, the changes in social interaction resulted in delayed onset of specific behaviors known as play behavior and in the maintenance of these behaviors until a period of adulthood in which they should have disappeared. These changes in social interaction in malnourished rats can be explained as a delayed development and it is suggested that they are directly related to delays in some aspects of maturation of the central nervous system.

Even after a period of nutritional rehabilitation, rats malnourished early in life may show behavioral changes relevant to adulthood and these changes may hamper the adaptation of animals to the environment in adult life. In addition to changes in typical behaviors of social interaction, malnourished rats have learning difficulties in tasks that involved complex strategies or required the animal’s attention to many environmental stimuli, as shown in previous studies by our group [31,32,34,46].

The malnutrition technique used in most of our studies consists in treatment with low-protein diets (6% protein) or protein-calorie malnutrition (50% restriction of the total amount of balanced diet offered to well-nourished groups) from the day of pups birth until the weaning or, in some cases, until 49 days of age, to access the effects of severe and long-lasting nutritional insults.

Fukuda *et al.* [31] studied spatial learning and memory processes in rats malnourished throughout the lactation period and after weaning until 49 days of age in two different spatial learning tasks. Animals were tested in proximal and distal cue versions of the Morris water maze. Well-nourished animals were provided with an adequate and balanced diet (16% protein) and malnourished ones were fed a low protein diet (6% protein). Animals from both groups received lab chow diet from 49 days of age until the end of the experiments (102 days of age). The period from 50 to 70 days of age was considered as a nutritional recovery phase for malnourished animals. The authors found no impairment of learning or memory processes of malnourished rats tested in the proximal cue version of the Morris water-maze but an increased latency and distance traveled to find the submerged platform in the distal cue version of the procedure.

The navigation in the ‘distal cue’ version is based in the relation among the rat, the goal and the extra-maze visual cues, suggesting that learning is mediated by the hippocampal formation. Malnourished rats spent more time swimming in circles or scanning the maze wall during the first trials of the test, in contrast with well-nourished animals that were faster in extinguishing this pattern of behavior and orienting the navigation in the direction of the center of the maze. The authors suggested that the present results can be due to alterations produced by protein malnutrition in the hippocampal formation or also to reflect the higher emotionality of rats following early malnutrition, especially considering the fact that postnatal malnourished rats are more reactive to unpleasant or aversive stimuli as cold water. It is also suggested that early malnutrition could produce behavior rigidity in the animals, impairing the extinction of behaviors that is not efficient to escape from the cold water (swimming by the maze wall) and this prolonging this behavior in detriment of others more efficient directed to the center of the water-maze where the escape platform is localized.

In another study conducted later by Fukuda and colleagues [46] the consequences of early postnatal malnutrition for the functioning the cholinergic system in learning task in the Morris water-maze after treating the animals with scopolamine by intraperitoneal (i.p.) injection was investigated. Scopolamine was administered before or after the sessions to verify possible learning and memory impairments. When scopolamine was administered before the trial sessions, there was a significant effect of the drug with learning impairments in both nutritional conditions. In the saline condition, well-nourished rats presented a better performance when compared with malnourished ones, but 28 days later, both groups increased their latencies in the memory retention trial. When scopolamine was administered after the trials sessions malnourished rats were also less reactive to the effects of the drug, resulting in lower impairments as compared to well-nourished rats. The results presented in this study, in agreement with those reported by Castro and Rudy [47], suggested that early protein malnutrition may produce long-term changes in memory and spatial learning processes as the result of enduring alterations in the hippocampal cholinergic system.

In 2003, Li-Tung Huang *et al.* [48] investigated postnatal malnutrition (using the technique of large litters) and/or seizure in the developing brain as a cause of hippocampal damages. Malnutrition procedure was imposed by culling rat pups to 20–22 pups per dam in malnourished group as compared with normal nourished rats with 10 pups per litter. Rats of each group were randomly assigned to either seizure or nonseizure group. Status epilepticus was induced by intraperitoneal (i.p.) injection of lithium chloride at postnatal day 20 and 60 mg/kg pilocarpine at postnatal day 21. The results demonstrated that in adulthood (postnatal day 80), both the seizure and malnourished groups showed spatial learning deficits, hippocampal cell loss and decreased level of phosphorylation of CREB^Ser133^ level within hippocampal CA1 region. Although the malnourished group demonstrated decreased levels of pCREB^Ser133^, no distinguishable changes in the cognitive deficit and hippocampal neuronal loss were detected.

Another study about the effects of malnourishment and seizures on memory and spatial learning in a model of developing brain was carried out by Hemb and colleagues [33]. Malnourished groups were maintained on a starvation regimen from postnatal day 2 (P2) to postnatal day 15 (P15). The undernourishment paradigm consisted of removing the dams from the cage starting at P2. The deprivation period was increased by 2 h for 6 consecutive days, from 2 h on P2 to 12 h on P7 and it remained at 12 h/day for the next 8 days (P8 to P15). During deprivation, pups remained in a light heated cage, with room temperature maintained at 34 °C. After the deprivation period, the pups were housed with their respective dams. Age-matched control rats remained with their dams. Seizure groups suffered three daily flurothyl-induced seizures from P2 to P4. The data indicated that early malnourishment does not alter seizure susceptibility at P15, but diminishes body and brain weight, whereas seizures diminish body but not brain weight. In the learning and memory test in the Morris water probe test it was observed that malnourished rats spent less time in the target quadrant than nourished animals, indicating learning impairment caused by malnutrition. The authors concluded that malnutrition and seizures have an additive detrimental effect on body and brain weight as well as on spatial memory.

Martínez and her co-workers [49] reported that learning and long-term retention of active avoidance test were impaired in young malnourished animals, but not in young control, senile control, and senile malnourished Sprague–Dawley rats; young and senile rats were 90 and 660 days of age, respectively. Pups in the control or well-fed group were born from dams fed with the 23.4% protein diet and fostered by other nurse dams fed with the 23.4% protein diet. The postnatal protein-malnourished group was composed of pups born from dams fed the 23.4% protein diet and then nursed by dams fed the 6% protein diet. Animals were fed either a 23.4% (well-nourished rats) or 6% protein diet (malnourished rats) until sacrifice at 92 or 662 days of age. Extinction tests showed that long-term memory of the malnourished groups and senile well-nourished rats was impaired as compared with the young well-nourished rats. The authors concluded that ageing and malnutrition were conditions capable to delay the maintenance of long-term memory, as seen during the extinction test.

A last study from our group [34] demonstrated that previously malnourished (6% protein) rats tested in three different learning and memory experiments (Morris water-maze—Experiment I, recognition memory of objects—Experiment II, and working memory in the water T-maze— Experiment III) showed higher escape latencies in Experiment I, lower recognition indexes in Experiment II and no differences due to diet in Experiment III. Well-nourished rats were fed a 16% protein diet. Our data suggested that protein malnutrition imposed on early life of rats can produce impairments on both working memory in the Morris maze and recognition memory in the open field tests.

The revised data above suggest that protein or protein-calorie malnutrition when imposed early in life is a significant biological variable that in conjunction with several environmental variables can lead to learning and memory impairments, depending on the severity and period of malnutrition paradigm imposed. Impairments in brain and body development as well in behavioral and cognition are the results of a malnutrition insult, and alterations in learning and memory is believed to reflect changes in hippocampal structures.

## 4. Discussion

The studies described above have shown that any change or manipulation capable to lead to severe or long-lasting malnutrition, early in life or during prenatal period, promotes changes known primarily as growth deficits and cognitive impairments, which are quite similar in humans and rodents (for more extensive reviews, see references [62–64]).

There are some possible explanations for the role that malnutrition in early life plays on cognitive development in both species. One is related to the critical period of brain development. During the development of central nervous system a sequential series of changes with a high degree of regulation occurs through chemical reactions, including cell division (neurogenesis and gliogenesis), migration of neurons and glial cells, cellular differentiation, myelinization, synapse formation, and synthesis and release of neurotransmitters. These sequences occur differently in various brain regions and even within a particular region. They also vary in time from one animal species to another, but do not vary fundamentally among mammals, though they are different in human and rodents with respect of the timing of birth in relation to the stage of brain maturation [62]. This consideration is of special consequence due to the use of animal models to examine brain processes affected by malnutrition. In humans, the critical period varies from the final third of gestation to the first 2 years of life; while in rats the critical period varies from the first week of gestation to the end of the third week of life. In that way, a nutritional insult during those periods has the potential to cause irreversible morphological, neurophysiological, neurochemical and functional damages [65].

One of the consequences of malnutrition during the critical period is a diffuse cortical involvement, which could explain some of the consequences previously described. For example, the right parietal cortex is related to visouspatial functions, and children’s poor performance on related tests [5] could be linked to its impairment. Like this, a number of cognitive deficits can be related to other cortical areas in both species.

Another structure of the brain particularly affected by early malnutrition in all mammals is hippocampus, which have been widely studied because it is directly involved with learning and memory processes [50,62,64,66]. The development of neurogenesis in the hippocampus occurs in the prenatal period of life and an extending period to postnatal life in both rats and humans [62]. Changes in hippocampal functioning, such as deficits in synaptic contact and neurotransmitter systems, that are believed to constitute the neurochemical bases for the processes of memory, can produce deficits in spatial learning and memory [46,50–52]. According to the theory of cognitive map or allocentric map proposed by O’Keefe and Nadel [67], the hippocampus is a structure in the central nervous system involved in learning processes and storage of spatial representation of the environment in cognitive maps. As postulated by these authors after several studies conducted in animals, the term cognitive map refers to the establishment of relations between cues associated with a determined place and its relative positions; this process results in the construction of spatial representations of the environment (cognitive maps) and enables the animal to use such representations to be guided in a specific spatial context. Through the inspection, manipulation or movements of locomotion in the environment (behaviors that constitute the animals’ exploration), information is acquired, processed and result in some kind of cognitive representation of the environment. Malnourished rats in early life are slower to exhibit performance similar to well-nourished ones in spatial navigation tasks and have less precise navigation strategies in the acquisition phase of learning. A widely used model for studying the processes of learning and spatial memory in malnourished rats is the Morris water-maze (for a more extensive review see [68]).

Although some studies do not show significant differences in performance between well-nourished and early malnourished rats in spatial learning and memory tasks [53–55]; some other studies with rats subjected to severe malnutrition in early life and/or extended until after the lactation period, covering thus the entire critical period of central nervous system development, showed that these animals take longer to learn the task and have more difficulty to retain the information learned [31,32,34,46,49,56,57].

When we assessed the studies conducted in recent decades, some general considerations can be made: (a) malnourished rats usually require more time to learn the task in learning tests; (b) when they learn, they take longer to extinguish the behavior, especially if the task involves food reward delivered as a consequence of the behavior; (c) malnutrition promotes an increased emotional reactivity to aversive or painful events and in situations where the animal’s performance depends on complex or subtle attentional processes. These behavioral changes observed in malnourished animals are explained as resulting from behavioral rigidity or cognitive inflexibility, characteristic of organisms malnourished during critical periods of central nervous system development and consists of difficulties in adaptation or processing of information in complex or changeable contexts.

Overall, the changes produced by malnutrition are determined by the type of nutritional insult to which the organism is submitted, the severity of this insult, its duration and the period of life where it happens. Severe malnutrition during the beginning of life, during critical periods of central nervous system development, produces some significant and lasting behavioral changes observable in the course of a lifetime, as demonstrated in human and animals studies discussed in the present review. Among the most significant cognitive changes we find learning difficulties and memory deficits.

Taking these finds together, we can assume that, in fact, supplement programs beginning early in life may play a critical role in improving the brain development and hence cognitive functioning, as previously demonstrated. However, some studies also demonstrated that psychosocial/environmental stimulation can lead to more permanent gains than nutritional supplementation alone. While some authors argue that benefits provided by psychosocial stimulation programs are transient, others affirm that these interventions are more efficient to long term life than just nutritional supplementation, suggesting that environment plays a more important role than nutrition itself. The question then is how the environment affects the developmentof cognition?

The most plausible argument is related to the sensitive period of brain development, which can be observed in children, and that is a concept different of the concept of critical periods. The sensitive period is seen as offering a broader window during which the brain is sensitive to a particular type of stimulation and represents periods in development during which certain capacities are readily shaped, e.g., language development [69], attention [70] and other human skills.

In that way, a child living in an environment which is social and culturally deprived could develop several cognitive problems, which is very common to observe in most of developing countries. Some important environmental characteristics associated with children’s poor cognitive performance are poorly educated parents and absent father [71], family poverty [72], neighborhood conditions [73] and inadequate provision of appropriate materials and games to the child [74].

Another possible explanation is that malnourished children have insufficient energy to take advantage of opportunities for social contacts and learning processes [14]. There are some evidences that a child previously malnourished is significantly more apathetic, and explores his environment with less enthusiasm than a control child [75], leaving to the suggestion that an unstimulating home environment could exacerbates the effects of malnutrition.

The mechanism through which early malnutrition and environmental stimulation may interact to produce cognitive problems both in humans and animals seems to have a clear and dependent relation. Levitsky and Barnes [58] proposed the functional isolation hypothesis, which the main premise is that the cognitive deficit found in malnourished organisms is the result of both direct and indirect influences. The direct mechanism is related to all the previously cited changes caused by malnutrition in the central nervous system during the critical period of development. In that way, malnutrition could make the organism less capable of receiving and/or integrating information from the environment and interact with it. The indirect influence may be purely behavioral in nature, which means that malnutrition *per se* produces behavior inconsistent with information from the external environment necessary for optimum cognitive development. These authors affirm that in malnourished animals the behavior is primarily food-oriented, while in children, the behavior may be expressed as apathy and social isolation, like already demonstrated here.

In summary, there is overwhelming evidences that malnutrition, especially imposed in early life, has significant and lasting implications for the development of cognition both in humans and animals. Malnutrition is typically associated with social and cultural deprivation and it is very difficult to distinguish the influence of one from another. So, strategies for reducing the effects of child malnutrition should address both nutritional supplementation and psychosocial stimulation during the early life for improving cognitive functions. It is suggested that future studies on this area should be focused on the mechanisms through which malnutrition interferes in social and environmental interaction and causes cognitive deficits. Once that mechanisms are established then it will be possible the implementation of more efficient large-scale intervention programs.

Although this review had shown that, in fact, malnutrition affects cognitive development in humans and animals, there are a small number of studies in this area of research. The authors call attention to the need to expand the research in this field, taking into account the importance of the effects of early malnutrition on cognitive functioning of both animals and humans. Special attention should be directed to the efforts in developing experimental behavior models in humans that allow us to correlate with those already well developed and described in the literature of animal studies, especially with rodents that comprise the great majority of data showing impairments of early malnutrition on cognitive processes.

## Figures and Tables

**Table 1.** Description of the studies conducted with humans included in this review.

**Study authors**	**Year**	**Nutritional supplementation**	**Psychosocial stimulation**	**Cognitive tasks**	**Statistical significance**	**Age of children**	**Country of study population**
Galler *et al.* [2]	1987	No	No	WISC-R^1^; Piagetian tests of conservation	Yes*	11–18 years	Barbados
Galler *et al.* [3]	1990	No	No	11-plus examination; WISC-R^1^; Classroom behavior	Yes*	11–18 years	Barbados
Agarwal *et al.* [4]	1992	No	No	Gesell’s development schedule; Binet Kulsrestha Intelligence scale; Level of Cognitive Ability; Block-Sort test	Yes*	1–3 years	India
Kar *et al.* [5]	2008	No	No	NIMHANS neuropsychological battery for children	Yes*	5–10 years	India
Mendez *et al.* [6]	1999	No	No	Philippines Non-Verbal Intelligence test; Cognitive test; Level of schooling	Yes*	8 and 11 years	Philippines
Berkman *et al.* [7]	2002	No	No	WISC-R^1^	Yes*	9 years	Peru
Miranda *et al.* [8]	2007	No	No	Raven’s Colored Progressive Matrices–Special scale; Cancellation of letters and symbol tests; WCST^2^; Stanford-Binet Scale; The Bender Visual Motor Gestalt test; Rivermead Behavioral Memory test; Corsi Block and Digital Span test; BCPR^3^; Educational Performance test; Teacher Rating Behavior scale	Yes*	7–10 years	Brazil
Chavez *et al.* [9]	1975	Yes	No	Physical, mental, and social development	Yes^#^	0–18 months	Mexico
Pollitt *et al.* [10]	1993	Yes	No	Brazelton Neonatal Assessment; Composite Infant Scale; Psychoeducational and information-processing tests	Yes^‡^	0–7 years	Guatemala
Whaley *et al.* [12]	2003	Yes	No	Raven’s Colored Progressive Matrices; Verbal Meaning test; Arithmetic test	Yes^‡^	7 years	Kenya
Freeman *et al.* [13]	1977	Yes	No	Language facility; Short-term memory for numbers; Perceptual analysis	Yes^‡^	3–4 years	Guatemala
Freeman *et al.* [14]	1980	Yes	No	Language facility; Short-term memory for numbers; Perceptual analysis; Cognitive composite	Yes^‡^	3–7 years	Guatemala
McKay *et al.* [16]	1978	Yes	Yes	Understanding complex commands; Figure tracing; Picture vocabulary; Intersensory perception; Colors, numbers and letters; Use of prepositions; Block construction; Cognitive maturity; Sentence completion; Memory for sentences; Knox cubes; Geometric drawings; Arithmetic; Mazes; Information; Vocabulary; Block design; Digit memory; Analogies and similarities; Matrices; Visual classification	Yes^‡^	3–7 years	Colombia
Waber *et al.* [17]	1981	Yes	Yes	Griffiths test; Einstein scale	Yes^‡^	3 years	Colombia
Grantham-McGregor *et al.* [18]	1991	Yes	Yes	Griffiths mental development scales	Yes^#^	9–24 months	Jamaica
Grantham-McGregor *et al.* [19]	1997	Yes	Yes	WRAT^4^; Stanford Binet test; PPVT^5^; Raven’s Progressive Matrices; Categorical fluency; Verbal analogies; Free call; French learning test; Digit span; Corsi blocks; Lafayette Grooved Pegboard test	Yes^#^	7–8 years	Jamaica
Walker *et al.* [20]	2000	Yes	Yes	Test of general intelligence; WISC-R^1^; test of visual reasoning ability; Raven’s Progressive Matrices; test of language comprehension; PPVT^5^; test of verbal analogies; Vocabulary test; test of auditory working memory; Digit span forwards and digit span backwards; test of visual-spatial memory; Corsi blocks; search test; Stroop test modified	Yes^#‡^	11–12 years	Jamaica
Walker *et al.* [21]	2005	Yes	Yes	WAIS^6^; Raven’s Progressive Matrices; Corsi blocks; Digit span forwards and digit span backwards; test of verbal analogies; PPVT^5^; group reading test 2-revised; WRAT^7^	Yes^#‡^	17–18 years	Jamaica

1 Wechsler Intelligence Scale for Children—Revised;

2 Wisconsin Card Sorting Test;

3 Brazilian Test of Pseudoword Repetition;

4 Range Achievement Test;

5 Peabody Picture Vocabulary test;

6 Wechsler adult intelligence scales;

7 Wide-range achievement test;

* Statistical significance on the comparison of malnourished and control children;

# Statistical significance on the comparison of treated and no treated children;

‡ Statistical significance on the comparison of different types of intervention.

**Table 2.** Description of the studies conducted with rodents included in this review.

**Study authors**	**Year**	**Nutritional rehabilitation**	**Cognitive tasks**	**Statistical significance**	**Age of animals**
Brioni & Orsingher [22]	1988	After weaning	Operant Reinforcement Test	Yes	70 days
Tonkiss, Shultz & Galler [23]	1994	After birth	Morris Water Maze (a) Distal-cue test (b) Proximal-cue test	No	16, 20, 70, 200–221 days
De Oliveira & Almeida [24]	1985	After weaning	Avoidance Performance Test	Yes	70 days
Almeida, de Oliveira & Graeff [25]	1990	After the 50th day of life	Hypertonic Water Ingestion and dose effect curves for (a) diazepam, (b) ipsapirone, (c) ritanserin and (d) isamoltane	Yes (a and b) No (c and d)	91 days
Almeida, de Oliveira & Graeff [26]	1991	After the 50th day of life	Elevated Plus-maze Test and Reactivity to Anxiolytic Drugs	Yes	70 days
Rocinholi, Almeida & De-Oliveira [27]	1997	After weaning	Shock Threshold Test (a) Tail-flick Test (b)	Yes (a) No (b)	70 days
Hernandes & Almeida [28]	2003	After the 50th day of life	Elevated T-maze (ETM) and Stretched Attend Posture (SAP)	Yes	70 days
Barnes *et al.* [29]	1966	After weaning (a) or after the 77th day of life (b)	Y Water Maze—Visual discrimination performance	Yes (b) No (a)	180–270 days
Tonkiss & Galler [30]	1990	Postnatal life	Elevated T-maze	Yes	90 or 160 days
Fukuda, Françolin-Silva & Almeida [31]	2002	After the 50th day of life	Morris Water Maze (a) Distal-cue test (b) Proximal-cue test	Yes (a) No (b)	70–77 and 102 days
Valadares & De Sousa Almeida [32]	2005	After weaning	Morris Water Maze (a) Spaced trials (b) Intermediate trials (c) Condensed trials	Yes (a) No (b and c)	70–77 and 102 days
Hemb, Cammarota & Nunes [33]	2010	---	Morris Water Maze (a) Undernutrition (b) Seizures	Yes	21–25 days
Valadares *et al.* [34]	2010	After weaning	Morris Water Maze (a) Recognition Memory of Objects (b) Water T-maze (c)	Yes (a and b) No (c)	70 days
Zhang, Li & Yang [35]	2010	After weaning	Morris Water Maze	Yes	70 days
Crnic & Chase [36]	1978	---	Milk Composition	Yes	---
Pine, Jessop & Oldham [37]	1994	---	Milk Composition	Yes	---
Passos, Ramos & Moura [38]	2000	---	Milk Composition	Yes	---
Hernandes *et al.* [39]	2005	After weaning	Elevated T-maze	Yes	70 days
Smart & Preece [40]	1973	---	Maternal Behavior	Yes	---
Crnic [41]	1980	---	Maternal Behavior	Yes	---
Riul *et al.* [42]	1999	---	Mother-pup Interaction	Yes	---
Lima *et al.* [43]	1993	After weaning	Lab Chow and Experimental Casein Diets Ingestion	Yes	49 days
Rocinholi, de Oliveira & Colafêmina [44]	2001	After weaning half the malnourished group	Brainstem Auditory Evoked Potentials	Yes	14, 18, 22, 32, and 42 days.
Camargo, Nascimento & Almeida [45]	2005	After weaning	Open Field—Social play behavior	Yes	26, 36, 46, 56, 66 and 76 days
Fukuda *et al.* [46]	2007	After the 50th day of life	Morris Water Maze after treatment with scopolamine (amnesic drug)	Yes	70 days
Castro & Rudy [47]	1993	After weaning	Conditional-spatial Discrimination Task		90 days
Huang *et al.* [48]	2003	After weaning	Morris Water Maze	No	80 days
Martínez *et al.* [49]	2009	After weaning	Active Avoidance Task	Yes	90 or 660 days
Lukoyanov & Andrade [50]	2000	From the 8th to the 10th month*	Open Field (a) Passive Avoidance Task (b) Morris Water Maze (c)	Yes (a and c) No (b)	10 months
Cintra *et al.* [51]	1990	---	Neuroanatomical Study	Yes	30, 90 and 220 days
Bedi [52]	1991	After the 30th day of life	Neuroanatomical Study	Yes	70 and 212 days
Wolf *et al.* [53]	1986	After weaning	Open Field (a) 8-arm Radial Maze (b)	Yes (a and b)	90 days
Campbell & Bedi. [54]	1989	After the 30th or the 60th day of life	Morris Water Maze	No	70 days
Bedi [55]	1992	After the 30th day of life	Morris Water Maze	Yes	35–65 or 170–200 days
Castro, Tracy & Rudy [56]	1989	After weaning	Conditional-spatial Discrimination Task-Win-shift version	Yes	23, 30, 40, and 90 days
Jordan, Cane & Howells [57]	1981	After weaning	8 and 16-arm Radial Maze	Yes	90 days
Levitsky & Barnes [58]	1972	After the 7th week of life	Social Behavior	Yes	Birth–17 weeks

* Malnutrition starting at 2 months of age, animals were maintained on low-protein diet either for 8 months, or for 6 months followed by a 2-month period of nutritional rehabilitation.
